# Coffee with a high content of chlorogenic acids and low content of hydroxyhydroquinone improves postprandial endothelial dysfunction in patients with borderline and stage 1 hypertension

**DOI:** 10.1007/s00394-018-1611-7

**Published:** 2018-01-12

**Authors:** Masato Kajikawa, Tatsuya Maruhashi, Takayuki Hidaka, Yukiko Nakano, Satoshi Kurisu, Takeshi Matsumoto, Yumiko Iwamoto, Shinji Kishimoto, Shogo Matsui, Yoshiki Aibara, Farina Mohamad Yusoff, Yasuki Kihara, Kazuaki Chayama, Chikara Goto, Kensuke Noma, Ayumu Nakashima, Takuya Watanabe, Hiroshi Tone, Masanobu Hibi, Noriko Osaki, Yoshihisa Katsuragi, Yukihito Higashi

**Affiliations:** 1 Division of Regeneration and Medicine, Medical Center for Translational and Clinical Research, Hiroshima University Hospital, Hiroshima, Japan; 2 Department of Cardiovascular Medicine, Graduate School of Biomedical and Health Sciences, Hiroshima University, Hiroshima, Japan; 3 Department of Cardiovascular Regeneration and Medicine, Research Institute for Radiation Biology and Medicine (RIRBM), Hiroshima University, 1-2-3 Kasumi, Minami-ku, Hiroshima, 734-8551 Japan; 4 Department of Gastroenterology and Metabolism, Graduate School of Biomedical and Health Sciences, Hiroshima University, Hiroshima, Japan; 5 Department of Physical Therapy, Hiroshima International University, Hiroshima, Japan; 6 Health Care Food Research Laboratories, Kao Corporation, Tokyo, Japan

**Keywords:** Chlorogenic acids, Hydroxyhydroquinone, Atherosclerosis, Endothelial function

## Abstract

**Purpose:**

The purpose of this study was to evaluate acute effects of coffee with a high content of chlorogenic acids and different hydroxyhydroquinone contents on postprandial endothelial dysfunction.

**Methods:**

This was a single-blind, randomized, placebo-controlled, crossover-within-subject clinical trial. A total of 37 patients with borderline or stage 1 hypertension were randomized to two study groups. The participants consumed a test meal with a single intake of the test coffee. Subjects in the Study 1 group were randomized to single intake of coffee with a high content of chlorogenic acids and low content of hydroxyhydroquinone or coffee with a high content of chlorogenic acids and a high content of hydroxyhydroquinone with crossover. Subjects in the Study 2 group were randomized to single intake of coffee with a high content of chlorogenic acids and low content of hydroxyhydroquinone or placebo coffee with crossover. Endothelial function assessed by flow-mediated vasodilation and plasma concentration of 8-isoprostanes were measured at baseline and at 1 and 2 h after coffee intake.

**Results:**

Compared with baseline values, single intake of coffee with a high content of chlorogenic acids and low content of hydroxyhydroquinone, but not coffee with a high content of chlorogenic acids and high content of hydroxyhydroquinone or placebo coffee, significantly improved postprandial flow-mediated vasodilation and decreased circulating 8-isoprostane levels.

**Conclusions:**

These findings suggest that a single intake of coffee with a high content of chlorogenic acids and low content of hydroxyhydroquinone is effective for improving postprandial endothelial dysfunction.

**Clinical Trial Registration:**

URL for Clinical Trial: https://upload.umin.ac.jp; Registration Number for Clinical Trial: UMIN000013283.

## Introduction

Endothelial dysfunction occurs in the early stage of atherosclerosis and plays an important role in the development of atherosclerotic conditions, resulting in cardiovascular complications [1, 2]. Measurements of flow-mediated vasodilation (FMD) as an index of endothelium-dependent vasodilation in the brachial artery have been widely used in clinical research to evaluate endothelial function [3–6]. Endothelial dysfunction has been shown to be an independent predictor of cardiovascular events [7–10]. Postprandial hyperglycemia is associated with endothelial dysfunction and is a risk factor for cardiovascular events [11, 12]. Acute hyperglycemia induces oxidative stress, which is a key trigger of endothelial dysfunction by reducing nitric oxide (NO) bioavailability [11, 12]. Therefore, it is important to determine interventions that can restore endothelial function under the condition of postprandial hyperglycemia.

Coffee is a popular beverage that is consumed worldwide. Coffee contains an abundance of polyphenols, which is the major source of dietary antioxidants [13, 14]. Drinking coffee is associated with lower risks of metabolic syndrome, diabetes, and coronary heart disease [15–19]. However, there have been conflicting results regarding the association between coffee drinking and risk of cardiovascular disease [19–23]. The effect of coffee on endothelial function is also controversial [24–27]. One possible reason for the different results of studies is that the contents of chlorogenic acids, which are the most abundant antioxidants in coffee, may vary depending on several factors [16]. Coffee has a complex chemical mixture with hundreds of compounds. Roasting coffee results in the loss of chlorogenic acids and generation of hydroxyhydroquinone [28]. A previous study showed that hydroxyhydroquinone inhibits the chlorogenic acid-induced restoration of endothelial function in a rat model of hypertension [29]. It has also been shown that hydroxyhydroquinone increased the production of reactive oxygen species in a dose-dependent manner [30]. Some clinical studies have shown that coffee with a high content of chlorogenic acids and a low content of hydroxyhydroquinone is effective for reducing blood pressure [31, 32].

However, there is no information on the effects of a combination of chlorogenic acids and hydroxyhydroquinone on endothelial function in humans. Therefore, in this study, we evaluated acute effects of coffee with a high content of chlorogenic acids and different contents of hydroxyhydroquinone on endothelial function, especially postprandial endothelial dysfunction, in patients with borderline or stage 1 hypertension.

## Materials and methods

### Subjects

Between October 2014 and January 2016, we enrolled 37 patients with borderline or stage 1 hypertension at Hiroshima University Hospital. The inclusion criteria were as follows: (1) patients with borderline or stage 1 hypertension, (2) 30 years of age or older, (3) non-smoker, and (4) alcohol intake ≤ 20 g/day. The exclusion criteria were as follows: (1) patients with diabetes mellitus, (2) treatment with renin angiotensin system inhibitors or statins, and (3) premenopausal women. It is well known that RAS inhibitors, but not calcium channel blockers, significantly influence the value of FMD [33]. Therefore, subjects on calcium channel blockers were not excluded in this study. None of participants took any medications other than calcium channel blockers. Borderline hypertension was defined as systolic blood pressure of 130–139 mmHg or diastolic blood pressure of 85–89 mmHg. Stage 1 hypertension was defined as systolic blood pressure of 140–159 mmHg or diastolic blood pressure of 90–99 mmHg [34]. Diabetes mellitus was defined according to the American Diabetes Association [35]. Dyslipidemia was defined according to the third report of the National Cholesterol Education Program [36]. This study was approved by the ethical committees of Hiroshima University. All subjects gave written informed consent for participation in the study.

### Study protocol

**Fig. 1:**
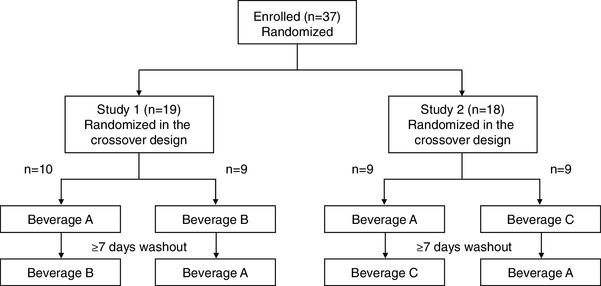
Flow chart of study design

This was a single-blind, randomized, placebo-controlled, crossover-within-subject clinical trial. A total of 37 subjects were randomized to Study 1 group or Study 2 group (Fig. 1). Study 1 group was randomized to single intake of beverage A (chlorogenic acids: 412 mg, hydroxyhydroquinone: 0.11 mg, and caffeine: 69 mg) or beverage B (chlorogenic acids: 373 mg, hydroxyhydroquinone: 0.76 mg, and caffeine: 75 mg) with crossover. Study 2 group was randomized to single intake of beverage A or beverage C (chlorogenic acids: 0 mg, hydroxyhydroquinone: 0.1 mg, and caffeine: 59 mg) with crossover. Each study was separated by a washout period of at least 7 days. The scientific conduct of the study (design, implementation, analysis and interpretation of the data) and manuscript preparation were independent of the sponsor. The subjects were instructed to avoid alcohol, coffee, and food containing chlorogenic acids for 24 h before the study, to abstain from eating for 12 h before the study and to drink only commercially available bottled water for 24 h before the study. The study began at 8:30 AM. Measurement of FMD was performed while each subject was in the supine position in a quiet, dark, air-conditioned room (constant temperature of 22–25 °C). Venous blood samples were obtained from the left antecubital vein. The subjects then ingested a test meal (592 kcal/115 g, carbohydrate:fat:protein = 75.0:28.5:8.0 g, Meal Test C; Saraya Co, Ltd, Osaka, Japan) with a single intake of a test beverage within 12 min. Blood samples were collected and FMD was measured at 1 and 2 h after ingestion. Chlorogenic acids in plasma were measured by liquid chromatography–tandem mass spectrometry. Plasma 8-isoprostane levels were measured using an EIA kit (Cayman Chemical Co., Ann Arbor, MI, USA).

### Test beverages

Ordinary coffee contains variable amounts of chlorogenic acids depending on the coffee bean species, brewing conditions, and roasting method. Darker roasts contain a smaller amount of chlorogenic acids [16]. Hydroxyhydroquinone is a compound that is generated from the roasting of coffee beans [28]. Direct measurements of commercially available brewed coffee showed that less than 200 mg chlorogenic acids is generally contained in one serving [37]. The beverages were manufactured industrially (Kao Co. Tokyo, Japan) and were taste- and flavor-matched (coffee-flavored). The compositions of the test beverages are shown in Table 1. Beverage A was roasted coffee for which an absorption purification process was used to remove hydroxyhydroquinone by active carbon, maintaining a large amount of chlorogenic acids. Beverage B was ordinary brewed coffee containing chlorogenic acids and hydroxyhydroquinone. Beverage C was an artificial placebo beverage prepared by using coffee-flavored agents.

**Table 1:** Composition of the intervention products

	Beverage A	Beverage B	Beverage C
Drink volume, g/serving	185	185	185
Chlorogenic acids (9 analogues), mg/serving	412	378	0
Hydroxyhydroquinone, mg/serving	0.11	0.76	0.10
Caffeine, mg/serving	69	75	59
Energy, kcal/serving	9	11	2

Chlorogenic acids (9 analogues) analogues present are the caffeoylquinic acids (3-CQA, 4-CQA, and 5-CQA), feruloylquinic acids (3-CQA, 4-CQA, and 5-CQA), and dicaffeoylquinic acids (3,4-di-CQA, 3,5-di-CQA, and 4,5-di-CQA)

### Measurement of FMD

A high-resolution ultrasonography (UNEXEF18G, UNEX Co, Nagoya, Japan) was used to evaluate FMD. The protocol for measurements of FMD have been described in detail previously [38]. Briefly, the longitudinal image of the brachial artery was assessed before and after generation of vascular response to reactive hyperemia by a 5-min period of forearm occlusion to evaluate FMD. FMD was defined as the maximal percentage change in vessel diameter from the baseline value.

### Statistical analysis

Results are presented as means ± SD for continuous variables and as percentages for categorical variables. Statistical significance was set at a level of *P* < 0.05. Comparison of continuous variables between 2 groups was performed using the Student’s t test or the Chi-squared test for categorical data. Changes in FMD and parameters between before and after drinking test beverages were evaluated using paired t test. Differences in FMD and parameters between beverage A and beverage B, and beverage A and beverage C were evaluated using repeated measures ANOVA with Tukey’s post hoc test. Relations between variables were determined by Pearson’s correlation analysis. The data were processed using the software package Stata version 9 (Stata Co., College Station, Texas, USA).

## Results

### Clinical characteristics

Baseline characteristics of Study 1 group and Study 2 group are summarized in Table 2. Baseline characteristics in the two groups were similar. All participants reported that they adhered to dietary restrictions, which was confirmed by low concentrations of plasma chlorogenic acids at baseline (Table 3). All participants completed the trial.

**Table 2:** Clinical characteristics of the subjects

Variables	Study 1 (*n* = 19)	Study 2 (*n* = 18)
Age, year	53 ± 19	56 ± 15
Sex, men/women	14/5	12/6
Body mass index, kg/m^2^	24.5 ± 4.1	23.2 ± 3.1
Systolic blood pressure, mmHg	130 ± 11	128 ± 13
Diastolic blood pressure, mmHg	77 ± 9	81 ± 8
Heart rate, bpm	65 ± 8	63 ± 9
HbA1c, %	5.4 ± 0.3	5.5 ± 0.4
Medical history, *n* (%)		
Borderline hypertension	6 (31.6)	3 (16.7)
Stage 1 hypertension	13 (68.4)	15 (83.3)
Dyslipidemia	9 (47.4)	7 (38.9)
Diabetes mellitus	0 (0)	0 (0)
Previous coronary heart disease	0 (0)	0 (0)
Previous stroke	0 (0)	0 (0)
Current smoker	0 (0)	0 (0)
Medications, *n* (%)		
Calcium-channel blockers	7 (36.7)	9 (50.0)
Renin angiotensin system inhibitors	0 (0)	0 (0)
Statins	0 (0)	0 (0)
Medically treated diabetes		
Any	0 (0)	0 (0)
Insulin-dependent	0 (0)	0 (0)

Results are presented as mean ± SD or number (%)

**Table 3:** FMD values at baseline and during follow-up

	Study 1		Study 2	
	Beverage A	Beverage B	Beverage A	Beverage C
Systolic blood pressure, mmHg				
Baseline	130 ± 11	131 ± 11	128 ± 13	130 ± 14
1 h	131 ± 13	128 ± 11	129 ± 12	132 ± 16
2 h	130 ± 12	129 ± 13	128 ± 16	129 ± 16
Chlorogenic acid, ng/mL				
Baseline	0.7 ± 2.0	1.1 ± 3.0	1.1 ± 2.7	0.7 ± 2.8
1 h	54.0 ± 20.5*	56.5 ± 22.7*	51.5 ± 22.5*	0.3 ± 1.3†
2 h	50.3 ± 16.0*	55.1 ± 23.3*	56.7 ± 21.2*	0.3 ± 1.2†
Triglycerides, mg/dL				
Baseline	124 ± 63	117 ± 65	121 ± 67	111 ± 49
1 h	159 ± 76*	143 ± 72*	140 ± 66*	132 ± 48*
2 h	181 ± 90*	188 ± 104*	174 ± 74*	167 ± 64*
Glucose, mg/dL				
Baseline	95 ± 10	93 ± 6	92 ± 8	92 ± 7
1 h	140 ± 32*	139 ± 32*	124 ± 23*	130 ± 22*
2 h	120 ± 29*	122 ± 24*	112 ± 16*	110 ± 18*
Insulin, µIU/L				
Baseline	7 ± 5	6 ± 4	6 ± 4	6 ± 3
1 h	55 ± 39*	51 ± 36*	42 ± 39*	42 ± 27*
2 h	41 ± 28*	45 ± 46*	26 ± 22*	27 ± 25*
8-Isoprostane, pg/mL				
Baseline	75 ± 48	55 ± 23	88 ± 67	73 ± 57
1 h	56 ± 41	59 ± 46	75 ± 67*	55 ± 23
2 h	73 ± 60	69 ± 74	72 ± 59*	64 ± 32
High-sensitivity C-reactive protein, mg/dL				
Baseline	0.10 ± 0.17	0.05 ± 0.04	0.07 ± 0.07	0.08 ± 0.17
1 h	0.10 ± 0.17	0.05 ± 0.04	0.07 ± 0.07	0.09 ± 0.21
2 h	0.10 ± 0.16	0.05 ± 0.04	0.07 ± 0.07	0.11 ± 0.25
FMD, %				
Baseline	4.5 ± 4.0	5.4 ± 4.8	3.1 ± 3.6	4.2 ± 3.6
1 h	5.4 ± 4.5*	4.9 ± 4.6	4.9 ± 2.1*	4.1 ± 3.3
2 h	6.0 ± 2.4*	6.4 ± 4.4	4.6 ± 3.4*	3.8 ± 3.7

*P < 0.05 vs. baseline

^†^*P* < 0.05 vs. Beverage A

### Effects of beverages on parameters and endothelial function

#### Study 1

Absolute changes in parameters after drinking test beverages are shown in Table 3. There were no significant differences in baseline and postprandial parameters at each observation time point between beverage A and beverage B (Table 3). The concentrations of triglycerides, glucose, insulin, and chlorogenic acid were significantly increased compared with baseline values at 1 h and at 2 h after ingestion of beverage A and after ingestion of beverage B. Single intake of beverage A significantly increased FMD from 4.5 ± 4.0% to 5.4 ± 4.5% at 1 h (an increase of 20%, *P* = 0.03) and to 6.0 ± 2.4% at 2 h (an increase of 33%, *P* = 0.04). FMD and 8-isoprostane were not different from baseline values after drinking beverage B. There were no significant differences in other postprandial parameters between beverage A and beverage B (Table 3).

#### Study 2

There were no significant differences in systolic blood pressure, triglycerides, glucose, insulin, 8-isoprostane, hs-CRP, and FMD at each observation time point between beverage A and beverage C (Table 3). The plasma chlorogenic acid concentrations were significantly higher after ingestion of beverage A than after ingestion of beverage C (Table 3). The concentrations of triglycerides, glucose, and insulin were significantly increased compared with baseline values at 1 and at 2 h after ingestion of beverage A and after ingestion of beverage C. Single intake of beverage A significantly increased FMD from 3.1 ± 3.6 to 4.9 ± 2.1% at 1 h (an increase of 58%, *P* = 0.02) and to 4.6 ± 3.4% at 2 h (an increase of 48%, *P* = 0.02) and significantly decreased serum level of 8-isoprostane from 88 ± 67 to 75 ± 67 pg/mL at 1 h (*P* = 0.03) and to 72 ± 59 pg/mL at 2 h (*P* = 0.02) (Table 3). FMD and 8-isoprostane were not different from baseline values after drinking beverage C. There were no significant differences in postprandial parameters between beverage A and beverage C (Table 3).

### Correlations between parameters and endothelial function

**Fig. 2:**
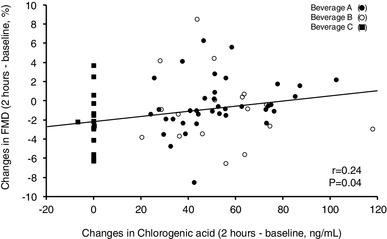
Scatter plot shows the relationships between postprandial changes in FMD and postprandial changes in chlorogenic acid

**Fig. 3:**
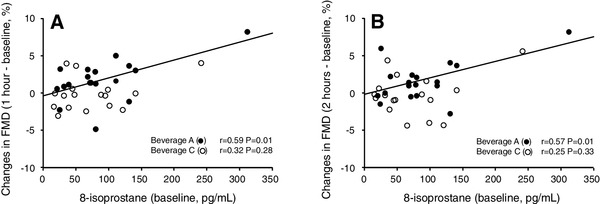
Scatter plots show the relationships between postprandial changes in FMD (**a** 1 h after ingestion, **b** 2 h after ingestion) and 8-isoprostane at baseline

Changes in FMD were positively correlated with changes in chlorogenic acids after drinking test beverages (Fig. 2). Changes in FMD after ingestion of beverage A were positively correlated with 8-isoprostane at baseline, while changes in FMD after ingestion of beverage C were not correlated with 8-isoprostane at baseline (Fig. 3a, b).

## Discussion

This study is the first single-blind, randomized, placebo-controlled, crossover trial to evaluate the acute effects of coffee with a high content of chlorogenic acids and different contents of hydroxyhydroquinone on postprandial endothelial dysfunction in patients with borderline or stage 1 hypertension. A single intake of coffee with a high content of chlorogenic acids and low content of hydroxyhydroquinone restored postprandial endothelial dysfunction by decreasing in oxidative stress.

Epidemiologic studies have shown that coffee drinking is associated with lower risk of cardiovascular disease [15, 17–19]. In the present study, single intake of coffee with a high content of chlorogenic acids and low content of hydroxyhydroquinone, but not single intake of coffee with a high content of chlorogenic acids and high content of hydroxyhydroquinone or placebo coffee, significantly improved postprandial endothelial dysfunction. In addition, changes in FMD after drinking the test beverage with a high content of chlorogenic acids and low content of hydroxyhydroquinone positively correlated with changes in levels of chlorogenic acids. These findings suggest that dietary intake of coffee with a high content of chlorogenic acids and low content of hydroxyhydroquinone is a healthy habit to improve endothelial function.

Oxidative stress has been shown to play a critical role in the maintenance and development of endothelial dysfunction by reducing the bioavailability of NO [1, 2]. Plasma levels of 8-isoprostane, a marker of oxidative stress, were increased in patients with vascular disease [39]. In the present study, single intake of coffee with a high content of chlorogenic acids and low content of hydroxyhydroquinone, but not single intake of coffee with a high content of chlorogenic acids and high content of hydroxyhydroquinone or placebo coffee, decreased circulating 8-isoprostane levels. In addition, coffee with a high content of chlorogenic acids and low content of hydroxyhydroquinone improved FMD in patients who had high levels of 8-isoprostane. These findings suggest that the beneficial effects of coffee with a high content of chlorogenic acids and low level of hydroxyhydroquinone on endothelial function are induced, at least in part, by a decrease in oxidative stress.

A major compound in coffee is caffeine. It is well known that caffeine influences systemic hemodynamics, including elevation of blood pressure and vascular function [40, 41]. Although both coffee with and without caffeine have similar associations with cardiovascular disease [15, 42], in the present study, to avoid the effects of caffeine on systemic hemodynamics and endothelial function, the test beverages contained the same amounts of caffeine.

A recent meta-analysis revealed antihypertensive effects of chlorogenic acids [43]. Suzuki et al. [29] reported that hydroxyhydroquinone inhibits the antihypertensive effect of chlorogenic acids in spontaneous hypertensive rats. These findings suggest that coffee with a high content of chlorogenic acids and low content of hydroxyhydroquinone has a beneficial effect in patients with hypertension. Indeed, long-term intake of coffee with a high content of chlorogenic acids and low content of hydroxyhydroquinone was shown to be effective for reducing blood pressure in patients with mild hypertension [31, 32]. Therefore, we enrolled patients with borderline or stage 1 hypertension in this study. In the present study, acute intake of coffee with a high content of chlorogenic acids and low content of hydroxyhydroquinone did not alter blood pressure in these subjects (Table 3). Several investigators, including us, have reported that caffeine intake is associated with acute increase in blood pressure [40, 41]. Conversely, chronic caffeine consumption was shown to have no significant effect on blood pressure [41]. In the case of acute coffee intake, interaction of chlorogenic acids and caffeine may regulate changes in blood pressure. It is likely that the antioxidant effect of chlorogenic acids, not the blood pressure-lowering effect of chlorogenic acids, is involved in the restoration of postprandial endothelial dysfunction.

Some studies have shown that acute administration of coffee has a harmful effect on endothelial function in healthy subjects [24, 25], while other studies have shown that coffee improves endothelial function [26, 27]. In addition, the association between coffee drinking and risk of cardiovascular disease remains inconclusive [15, 17–23]. The reason for the controversial results remains unclear. Hydroxyhydroquinone inhibits the chlorogenic acid-induced restoration of endothelial function and increases production of reactive oxygen species in a dose-dependent manner [29, 30]. To evaluate the effects of hydroxyhydroquinone on endothelial function, we compared the effects of coffees with a high content of chlorogenic acids and different contents of hydroxyhydroquinone in Study 1. We confirmed that intake of coffee with a high content of chlorogenic acids and low content of hydroxyhydroquinone, but not intake of coffee with a high content of chlorogenic acids and high content of hydroxyhydroquinone, improved postprandial endothelial dysfunction. Intake of coffee with reduced hydroxyhydroquinone per se also may be beneficial for maintenance of vascular function and prevention of cardiovascular events.

### Study limitations

The present study has a number of limitations. First, the number of patients was relatively small. A single-blind, randomized, placebo-controlled, crossover trial was performed to increase the power of the study. We confirmed that a single intake of coffee with a high content of chlorogenic acids and low content of hydroxyhydroquinone was effective for restoring postprandial endothelial dysfunction in both Study 1 and Study 2. Second, we evaluated the acute effects of chlorogenic acids and chlorogenic acids with different hydroxyhydroquinone contents on endothelial function. Long-term interventions are needed to determine whether acute effects of coffee with a high content of chlorogenic acids and low content of hydroxyhydroquinone are sustained over time. Third, coffee is a rich source of chlorogenic acids, which have strong anti-inflammatory properties [13, 14]. Inflammation also plays a critical role in endothelial dysfunction [44, 45]. In the present study, there were no significant differences in serum levels of high-sensitivity C-reactive protein between beverage A and beverage B (Table 3). It is unlikely that inflammation contributes to high content of chlorogenic acid-induced improvement in postprandial vascular injury. Fourth, we had no information on the participants’ coffee consumption background. Although we confirmed that the concentrations of plasma chlorogenic acids at baseline were low, we cannot deny the possibility that the participants’ coffee consumption background affected the results of the study. Finally, coffee contains hundreds of compounds that might affect endothelial function. We cannot rule out the possibility that compounds other than chlorogenic acids and hydroxyhydroquinone have a greater influence on endothelial function.

In conclusion, a single intake of coffee with a high content of chlorogenic acids and low hydroxyhydroquinone is effective for improving postprandial endothelial dysfunction. Further studies are needed to assess the long-term effects of drinking coffee with a high content of chlorogenic acids and low content of hydroxyhydroquinone on vascular function, onset of cardiovascular disease, and cardiovascular events.

## References

[1] Ross R (1999). Atherosclerosis-an inflammatory disease. N Engl J Med.

[2] Higashi Y, Noma K, Yoshizumi M, Kihara Y (2009). Oxidative stress and endothelial function in cardiovascular diseases. Circ J.

[3] Celermajer DS, Sorensen KE, Gooch VM, Spiegelhalter DJ, Miller OI, Sullivan ID, Lloyd JK, Deanfield JE (1992). Non-invasive detection of endothelial dysfunction in children and adults at risk of atherosclerosis. Lancet.

[4] Corretti MC, Anderson TJ, Benjamin EJ, Celermajer D, Charbonneau F, Creager MA, Deanfield J, Drexler H, Gerhard-Herman M, Herrington D, Vallance P, Vita J, Vogel R, International Brachial Artery Reactivity Task Force (2002). Guidelines for the ultrasound assessment of endothelial-dependent flow-mediated vasodilation of the brachial artery: a report of the International Brachial Artery Reactivity Task Force. J Am Coll Cardiol.

[5] Benjamin EJ, Larson MG, Keyes MJ, Mitchell GF, Vasan RS, Keaney JF, Lehman BT, Fan S, Osypiuk E, Vita JA (2004). Clinical correlates and heritability of flow-mediated dilation in the community: the Framingham Heart Study. Circulation.

[6] Kajikawa M, Oda N, Kishimoto S, Maruhashi T, Iwamoto Y, Iwamoto A, Matsui S, Aibara Y, Yusoff MF, Hidaka T, Kihara Y, Chayama K, Goto C, Noma K, Nakashima A, Taguchi A, Higashi Y (2017). Increasing risk of osteoporotic fracture is associated with vascular dysfunction and abnormal vascular structure in both men and women. Circ J.

[7] Modena MG, Bonetti L, Coppi F, Bursi F, Rossi R (2002). Prognostic role of reversible endothelial dysfunction in hypertensive postmenopausal women. J Am Coll Cardiol.

[8] Gokce N, Keaney JF, Hunter LM, Watkins MT, Menzoian JO, Vita JA (2002). Risk stratification for postoperative cardiovascular events via noninvasive assessment of endothelial function: a prospective study. Circulation.

[9] Lerman A, Zeiher AM (2005). Endothelial function: cardiac events. Circulation.

[10] Morimoto H, Kajikawa M, Oda N, Idei N, Hirano H, Hida E, Maruhashi T, Iwamoto Y, Kishimoto S, Matsui S, Aibara Y, Hidaka T, Kihara Y, Chayama K, Goto C, Noma K, Nakashima A, Ukawa T, Tsuji T, Higashi Y (2016). Endothelial function assessed by automatic measurement of enclosed zone flow-mediated vasodilation using an oscillometric method is an independent predictor of cardiovascular events. J Am Heart Assoc.

[11] Loader J, Montero D, Lorenzen C, Watts R, Méziat C, Reboul C, Stewart S, Walther G (2015). Acute hyperglycemia impairs vascular function in healthy and cardiometabolic diseased subjects: systematic review and meta-analysis. Arterioscler Thromb Vasc Biol.

[12] Mah E, Bruno RS (2012). Postprandial hyperglycemia on vascular endothelial function: mechanisms and consequences. Nutr Res.

[13] Gómez-Ruiz JA, Leake DS, Ames JM (2007). In vitro antioxidant activity of coffee compounds and their metabolites. J Agric Food Chem.

[14] Fukushima Y, Ohie T, Yonekawa Y, Yonemoto K, Aizawa H, Mori Y, Watanabe M, Takeuchi M, Hasegawa M, Taguchi C, Kondo K (2009). Coffee and green tea as a large source of antioxidant polyphenols in the Japanese population. J Agric Food Chem.

[15] Freedman ND, Park Y, Abnet CC, Hollenbeck AR, Sinha R (2012). Association of coffee drinking with total and cause-specific mortality. N Engl J Med.

[16] Martini D, Del Bo’ C, Tassotti M, Riso P, Del Rio D, Brighenti F, Porrini M (2016). Coffee consumption and oxidative stress: a review of human intervention studies. Molecules.

[17] van Dam RM, Feskens EJ (2002). Coffee consumption and risk of type 2 diabetes mellitus. Lancet.

[18] Hino A, Adachi H, Enomoto M, Furuki K, Shigetoh Y, Ohtsuka M, Kumagae S, Hirai Y, Jalaldin A, Satoh A, Imaizumi T (2007). Habitual coffee but not green tea consumption is inversely associated with metabolic syndrome: an epidemiological study in a general Japanese population. Diabetes Res Clin Pract.

[19] Ding M, Bhupathiraju SN, Satija A, van Dam RM, Hu FB (2014). Long-term coffee consumption and risk of cardiovascular disease: a systematic review and a dose-response meta-analysis of prospective cohort studies. Circulation.

[20] Sugiyama K, Kuriyama S, Akhter M, Kakizaki M, Nakaya N, Ohmori-Matsuda K, Shimazu T, Nagai M, Sugawara Y, Hozawa A, Fukao A, Tsuji I (2010). Coffee consumption and mortality due to all causes, cardiovascular disease, and cancer in Japanese women. J Nutr.

[21] Gardener H, Rundek T, Wright CB, Elkind MS, Sacco RL (2013). Coffee and tea consumption are inversely associated with mortality in a multiethnic urban population. J Nutr.

[22] Lopez-Garcia E, Rodriguez-Artalejo F, Li TY, Mukamal KJ, Hu FB, van Dam RM (2011). Coffee consumption and mortality in women with cardiovascular disease. Am J Clin Nutr.

[23] Grobbee DE, Rimm EB, Giovannucci E, Colditz G, Stampfer M, Willett W (1990). Coffee, caffeine, and cardiovascular disease in men. N Engl J Med.

[24] Papamichael CM, Aznaouridis KA, Karatzis EN, Karatzi KN, Stamatelopoulos KS, Vamvakou G, Lekakis JP, Mavrikakis ME (2005). Effect of coffee on endothelial function in healthy subjects: the role of caffeine. Clin Sci (Lond).

[25] Buscemi S, Verga S, Batsis JA, Donatelli M, Tranchina MR, Belmonte S, Mattina A, Re A, Cerasola G (2010). Acute effects of coffee on endothelial function in healthy subjects. Eur J Clin Nutr.

[26] Buscemi S, Verga S, Batsis JA, Tranchina MR, Belmonte S, Mattina A, Re A, Rizzo R, Cerasola G (2009). Dose-dependent effects of decaffeinated coffee on endothelial function in healthy subjects. Eur J Clin Nutr.

[27] Shechter M, Shalmon G, Scheinowitz M, Koren-Morag N, Feinberg MS, Harats D, Sela BA, Sharabi Y, Chouraqui P (2011). Impact of acute caffeine ingestion on endothelial function in subjects with and without coronary artery disease. Am J Cardiol.

[28] Gaascht F, Dicato M, Diederich M (2015). Coffee provides a natural multitarget pharmacopeia against the hallmarks of cancer. Genes Nutr.

[29] Suzuki A, Fujii A, Jokura H, Tokimitsu I, Hase T, Saito I (2008). Hydroxyhydroquinone interferes with the chlorogenic acid-induced restoration of endothelial function in spontaneously hypertensive rats. Am J Hypertens.

[30] Shashni B, Sharma K, Singh R, Sakharkar KR, Dhillon SK, Nagasaki Y, Sakharkar MK (2013). Coffee component hydroxyl hydroquinone (HHQ) as a putative ligand for PPAR gamma and implications in breast cancer. BMC Genom.

[31] Ochiai R, Chikama A, Kataoka K, Tokimitsu I, Maekawa Y, Ohishi M, Rakugi H, Mikami H (2009). Effects of hydroxyhydroquinone-reduced coffee on vasoreactivity and blood pressure. Hypertens Res.

[32] Yamaguchi T, Chikama A, Mori K, Watanabe T, Shioya Y, Katsuragi Y, Tokimitsu I (2008). Hydroxyhydroquinone-free coffee: a double-blind, randomized controlled dose-response study of blood pressure. Nutr Metab Cardiovasc Dis.

[33] Fujimura N, Noma K, Hata T, Soga J, Hidaka T, Idei N, Fujii Y, Mikami S, Maruhashi T, Iwamoto Y, Kihara Y, Chayama K, Kato H, Liao JK, Higashi Y (2012). Mineralocorticoid receptor blocker eplerenone improves endothelial function and inhibits rho-associated kinase activity in patients with hypertension. Clin Pharmacol Ther.

[34] James PA, Oparil S, Carter BL, Cushman WC, Dennison-Himmelfarb C, Handler J, Lackland DT, LeFevre ML, MacKenzie TD, Ogedegbe O, Smith SC, Svetkey LP, Taler SJ, Townsend RR, Wright JT, Narva AS, Ortiz E (2014). 2014 evidence-based guideline for the management of high blood pressure in adults: report from the panel members appointed to the Eighth Joint National Committee (JNC 8). JAMA.

[35] American Diabetes Association (2017). Classification and diagnosis of diabetes. Diabetes Care.

[36] Expert Panel on Detection, Evaluation, and Treatment of High Blood Cholesterol in Adults (2001). Executive Summary of the Third Report of the National Cholesterol Education Program (NCEP) Expert Panel on Detection, Evaluation, and Treatment of High Blood Cholesterol in Adults (Adult Treatment Panel III). JAMA.

[37] Ludwig IA, Mena P, Calani L, Cid C, Del Rio D, Lean ME, Crozier A (2014). Variations in caffeine and chlorogenic acid contents of coffees: what are we drinking?. Food Funct.

[38] Maruhashi T, Soga J, Fujimura N, Idei N, Mikami S, Iwamoto Y, Kajikawa M, Matsumoto T, Hidaka T, Kihara Y, Chayama K, Noma K, Nakashima A, Goto C, Higashi Y (2013). Nitroglycerine-induced vasodilation for assessment of vascular function: a comparison with flow-mediated vasodilation. Arterioscler Thromb Vasc Biol.

[39] Montuschi P, Barnes PJ, Roberts LJ (2004). Isoprostanes: markers and mediators of oxidative stress. FASEB J.

[40] Umemura T, Ueda K, Nishioka K, Hidaka T, Takemoto H, Nakamura S, Jitsuiki D, Soga J, Goto C, Chayama K, Yoshizumi M, Higashi Y (2006). Effects of acute administration of caffeine on vascular function. Am J Cardiol.

[41] Mesas AE, Leon-Muñoz LM, Rodriguez-Artalejo F, Lopez-Garcia E (2011). The effect of coffee on blood pressure and cardiovascular disease in hypertensive individuals: a systematic review and meta-analysis. Am J Clin Nutr.

[42] Ding M, Satija A, Bhupathiraju SN, Hu Y, Sun Q, Han J, Lopez-Garcia E, Willett W, van Dam RM, Hu FB (2015). Association of coffee consumption with total and cause-specific mortality in 3 large prospective cohorts. Circulation.

[43] Onakpoya IJ, Spencer EA, Thompson MJ, Heneghan CJ (2015). The effect of chlorogenic acid on blood pressure: a systematic review and meta-analysis of randomized clinical trials. J Hum Hypertens.

[44] Libby P, Ridker PM, Maseri A (2002). Inflammation and atherosclerosis. Circulation.

[45] Bhagat K, Vallance P (1997). Inflammatory cytokines impair endothelium-dependent dilatation in human veins in vivo. Circulation.

